# Hematological Predictors of Impaired Postpartum Uterine Involution in Thrombophilia: A Multivariate Analysis

**DOI:** 10.3390/diagnostics16030454

**Published:** 2026-02-01

**Authors:** Loredana Toma, Roxana Covali, Demetra Socolov, Alexandru Carauleanu, Mihaela Camelia Tirnovanu, Alin Ciubotaru, Laura Riscanu, Diana Lacatusu, Cristiana Filip

**Affiliations:** 1 Department of Obstetrics and Gynecology, Grigore T. Popa University of Medicine and Pharmacy, 700115 Iasi, Romania; loredana-toma@umfiasi.ro; 2 Elena Doamna Obstetrics and Gynecology University Hospital, 700398 Iasi, Romania; 3 Department of Radiology, Grigore T. Popa University of Medicine and Pharmacy, 700115 Iasi, Romania; 4 Department of Mother and Child, Grigore T. Popa University of Medicine and Pharmacy, 700115 Iasi, Romania; demetra.socolov@umfiasi.ro (D.S.); alexandru.carauleanu@umfiasi.ro (A.C.); mihaela.tirnovanu@umfiasi.ro (M.C.T.); 5 Cuza Voda Obstetrics and Gynecology University Hospital, 700038 Iasi, Romania; 6 Department of Neurology, Grigore T. Popa University of Medicine and Pharmacy, 700115 Iasi, Romania; alinciubotaru94@yahoo.com; 7 Department of Morphofunctional Sciences, Grigore T. Popa University of Medicine and Pharmacy, 700115 Iasi, Romania; laura.knieling@umfiasi.ro; 8 Department of Pharmaceutical Physics, Grigore T. Popa University of Medicine and Pharmacy, 700115 Iasi, Romania; diana.lacatusu@umfiasi.ro; 9 Department of Biochemistry, Grigore T. Popa University of Medicine and Pharmacy, 700115 Iasi, Romania; cristiana.filip@umfiasi.ro

**Keywords:** thrombophilia, pregnancy, postpartum uterine ultrasonographic scale, multivariate regression model, MLR, NLPR, IIC

## Abstract

**Background:** Although thrombophilia represents a major risk factor for adverse maternal outcomes, particularly in the postpartum period, methods for its systematic screening remain costly and limited. This case–control study aimed to evaluate whether routinely available hematological inflammatory indices combined with postpartum uterine ultrasonographic assessment can predict the presence of thrombophilia in peripartum women. **Methods:** Eighty women with previously diagnosed and treated thrombophilia undergoing cesarean section at term were prospectively enrolled and matched by age and parity with 80 control patients without thrombophilia. Hematological inflammatory markers derived from complete blood counts obtained within 24 h before delivery and the postpartum uterine ultrasonographic score were analyzed. Multivariable logistic regression was performed to identify independent predictors of thrombophilia, and odds ratios (ORs) with 95% confidence intervals (CIs) were calculated. **Results:** Impaired postpartum uterine involution—defined as a postpartum uterine ultrasonographic score ic—was significantly more frequent in thrombophilia cases than in controls (OR > 1, 95% CI excluding 1; *p* < 0.05). Thrombophilia patients exhibited significantly higher Neutrophil-to-Lymphocyte and Platelet Ratio and Cumulative Inflammatory Index values when compared with the controls, with both emerging as independent predictors in the multivariable model (OR > 1, 95% CI excluding 1; *p* < 0.05). The final model demonstrated good discriminative performance, with an overall classification accuracy of 88.6% and excellent specificity for excluding thrombophilia when the postpartum uterine ultrasonographic score was 0. **Conclusions:** The integration of postpartum uterine ultrasonographic assessment with simple hematological inflammatory indices provides a non-invasive, cost-effective approach for identifying women at increased risk of underlying thrombophilia in the immediate postpartum period. This strategy may support targeted thromboprophylaxis and rationalize the use of specialized thrombophilia testing.

## 1. Introduction

Thrombophilia refers to a tendency towards hypercoagulability caused by genetic or acquired hemostasis conditions [1]. Pregnancy itself favors hypercoagulability [2], which accelerates during pregnancy and reaches its highest point in the peripartum period [3]. Thrombophilia alters pregnancy outcomes due to hypercoagulability, stasis, and placental modifications [4,5,6,7,8].

The American Society of Hematology has issued conditional recommendations for thrombophilia testing in pregnant women with a family history of high-risk thrombophilia types [9]. However, an extensive workup of postpartum thrombophilia contributes only a low proportion of positive findings; therefore, testing for inherited thrombophilia should be reserved for patients with a personal or family history of thrombosis [10] or women with pregnancy-related morbidity [11]. Moreover, the cost of thrombophilia testing may be prohibitive for many at-risk pregnant patients. Therefore, while molecular diagnostic techniques are available, there remains a need for robust, cost-effective, and accurate screening methods that are applicable to large populations [12].

The PUUS was introduced to study the influence of a variety of factors on the speed of uterine involution [13]. In a previous study [14], we demonstrated that postpartum uterine involution in thrombophilia patients was correlated with postpartum neutrophil and platelet counts.

Elevated levels of immune inflammatory markers such as SII, SIRI, and LDH are strong predictors of adverse pregnancy outcomes in thrombophilia-complicated pregnancies [15]; in particular, these markers are significantly associated with negative maternal–neonatal outcomes [15], and thus, may play a role in predicting thrombophilia in pregnant patients who cannot afford direct testing.

Therefore, in this study we constructed and analyzed a model for predicting thrombophilia in patients, based on inflammation indices and the postpartum uterine ultrasonographic scale score.

Clinical Significance and Practical Implications

The identification of a statistically significant predictive model prompts the essential question: What is its tangible impact on clinical practice? The principal clinical significance of our findings lies in providing a cost-effective, accessible, and non-invasive screening tool for the immediate postpartum period. This tool can flag patients with a high probability of underlying, previously undiagnosed thrombophilia, enabling targeted prophylactic interventions that can alter both short- and long-term patient trajectories.

The practical implications of this model translate into a structured, actionable clinical pathway:

1. Immediate Postpartum Risk Stratification and Prophylaxis Triage

In current practice, the decision to administer postpartum thromboprophylaxis often relies on generic risk assessment scores (e.g., the Caprini score), which may not capture the specific risk conferred by occult thrombophilia. Our model refines this decision: a patient presenting with a PUUS score ≥ 1 in conjunction with elevated inflammatory indices (notably NLPR and IIC) falls into a newly defined high-risk category, with a model-indicated significant probability of thrombophilia. This finding provides a concrete, biomarker-supported rationale for the following:

- The immediate initiation of low-molecular-weight heparin (LMWH) thromboprophylaxis at prophylactic doses, extending through the high-risk puerperium (typically 6 weeks postpartum).
- Enhanced monitoring for signs of venous thromboembolism (VTE), such as deep vein thrombosis (DVT) or pulmonary embolism (PE), during hospital stay and in early follow-up.

2. Rationalizing Costly and Invasive Diagnostic Workups

Comprehensive thrombophilia testing is expensive, not universally accessible, and typically has low yield in unselected populations. In this context, our model acts as a pre-screening filter. For patients with a high predicted probability (e.g., PUUS ≥ 2, elevated NLPR/IIC), the model provides strong justification for proceeding with confirmatory molecular thrombophilia testing, ensuring that resources are directed where the pre-test probability is highest.

For patients with a low predicted probability (PUUS = 0 and normal inflammatory indices), the model suggests a very low likelihood of thrombophilia. This can prevent unnecessary testing, thus reducing healthcare costs and avoiding patient anxiety associated with false-positive or incidental findings from broad thrombophilia panels.

3. Informing Long-Term Management and Counseling

A prediction of thrombophilia postpartum has implications far beyond the immediate puerperium—it opens a critical window for patient education and future pregnancy planning.

Lifestyle and pharmacological management: Patients identified as high-risk can be counseled on lifelong thromboprophylactic measures, including the avoidance of combined oral contraceptives, the importance of hydration and mobilization during future pregnancies or prolonged immobility, and the potential need for LMWH prophylaxis in future high-risk situations (e.g., surgery).

Reproductive counseling: The model’s prediction provides a plausible explanation for previous adverse obstetric outcomes (e.g., recurrent pregnancy loss, fetal growth restriction) and informs the management of future pregnancies. Patients can be advised that in subsequent pregnancies, prophylactic or therapeutic LMWH may be recommended from the first trimester, potentially improving live birth rates and reducing placental-mediated complications.

4. Creating a Biomarker-Guided Follow-Up Protocol

The model integrates uterine imaging and hematological parameters into a unified risk assessment. This facilitates the establishment of a standardized follow-up protocol for high-risk patients:

- Postpartum ultrasound surveillance: Patients with elevated PUUS scores can undergo scheduled follow-up sonography to ensure complete uterine involution and rule out retained products of conception, which themselves are a risk factor for late postpartum hemorrhage and infection.
- Monitoring of inflammatory status: Serial complete blood counts could be used to track the normalization of inflammatory indices, providing dynamic feedback on the patient’s recovery and systemic inflammatory state.

Novelty and Original Contribution

This research presents various new and complementary aspects in the domains of postpartum care and thrombophilia risk evaluation. It primarily establishes, for the first time, a clear and measurable connection between delayed postpartum uterine involution (as objectively assessed via the PUUS score) and a systemic inflammatory-hematological profile indicative of thrombophilia. Although inflammatory indices have been examined in different contexts, combining them with a particular postpartum ultrasonographic finding to develop a composite predictive model is novel.

Additionally, the study recognizes the NLPR (Neutrophil-to-Lymphocyte and Platelet Ratio) and IIC (Cumulative Inflammatory Index) as notably significant predictors, emphasizing these unconventional ratios as powerful biomarkers in the postpartum thrombotic risk scenario. Ultimately, the main innovation of the study lies in its pragmatic, translational design: it advances from showing correlation to creating an easily applicable multivariate model utilizing affordable, commonly available parameters (complete blood count and ultrasound), thus providing a practical screening approach for environments with differing resources where extensive thrombophilia testing is unattainable. The combination of a new clinical imaging correlate, the confirmation of specific hematological inflammatory ratios, and the development of a practical predictive tool represents the unique and important contribution of this study.

The main patient outcome enabled by this predictive model is a transition from undiagnosed, high-risk condition to actively monitored, risk-reduced treatment. Rather than being conclusive, the prediction serves as a vital decision point that initiates a series of specific interventions, significantly changing the patient’s clinical path. The direct result is the prevention of potentially dangerous postpartum venous thromboembolism (VTE) via the warranted start of thromboprophylaxis in a formerly unrecognized group. As a result, this may reduce the incidence of deep vein thrombosis or pulmonary embolism and, thus, avoid related fatalities.

Patient Outcomes Following Thrombophilia Prediction

From a long-term perspective, the prediction of thrombophilia allows for educated family planning and enhanced obstetric outcomes. Patients recognized as having a strong likelihood of thrombophilia are provided essential counseling about the notably heightened risks of placental-related issues (e.g., recurrent pregnancy loss, preeclampsia, fetal growth restriction) in future pregnancies.

This information enables pre-conception preparation and the implementation of prophylactic low-molecular-weight heparin from the initial phases of the subsequent pregnancy, which has been shown to improve live birth rates. Moreover, patients develop a lifelong understanding of their thrombotic risk, enabling them to steer clear of high-risk estrogen-based contraceptives, manage future surgeries or times of immobilization proactively, and cultivate stronger health literacy. Consequently, the final result extends beyond the postpartum phase, promoting enhanced long-term health, safer subsequent pregnancies, and a decrease in the overall disease burden tied to undiagnosed thrombophilia.

## 2. Materials and Methods

This research utilized a prospective case–control framework alongside retrospective data examination, pairing 80 pregnant individuals with diagnosed, treated thrombophilia by age and parity with 80 control subjects. This design is particularly suitable for studying a condition (thrombophilia) that has a relatively low prevalence in the population, as it effectively facilitates the comparison of various exposures (inflammatory indices) between a specific case group and a matching control group. The statistical power is reinforced by the sample size of 160 participants, which offered adequate data for strong multivariate and Bayesian modeling.

Matching based on key demographic factors enhances internal validity by minimizing confounding. The retrospective examination of data collected prospectively (e.g., blood metrics and PUUS ratings) leverages the precision of standardized clinical protocols while allowing for the creation of intricate statistical models. As a result, this combined approach is particularly appropriate for achieving the study’s objective: it enables the detection and measurement of particular hematological and ultrasonographic characteristics linked to thrombophilia, thus permitting the creation of a predictive model based on easily obtainable clinical data, while avoiding the ethical and logistical issues of a randomized trial. Patients were referred to our hospital for delivery at term by means of cesarean section between 1 October 2017 and 1 December 2021. Hospital policy required that patients already diagnosed with thrombophilia were provided a cesarean section at 38 weeks gestational age, as were the study group patients. All thrombophilia patients already had an established diagnosis, and ongoing treatment with low-molecular-weight heparin. As our hospital cannot supply thrombophilia screening tests, the control group had their blood sent for screening to specialized laboratories, which yielded negative results. The exclusion criteria were as follows: patients suffering from thrombocytopenia (n = 2), patients with deep vein thrombosis (n = 0), and patients with cerebral thrombosis (n = 0) [14]. These prospectively gathered data were retrospectively used to construct a model.

The study was conducted in accordance with the Declaration of Helsinki and was approved by the Ethics Committee of Elena Doamna Obstetrics and Gynecology University Hospital (approval number 9 from 17 September 2017).

Every patient received a sonogram during the first 1–2 days after cesarean section, and the uterine evaluation was interpreted according to the Postpartum Uterine Ultrasonographic Scale (PUUS). This scale [13,14] counts the quarters of missing uterine vacuum lines, which could be due to blood or debris presence, as follows:

In grade 0, the uterine cavity is completely empty.

In grade 1, there is a small amount of blood or debris, occupying less than one-quarter of the vacuum line.

In grade 2, there is a slightly larger amount of blood or debris, occupying less than two-quarters of the vacuum line.

In grade 3, there is a large amount of blood or debris, occupying less than three-quarters of the vacuum line.

In grade 4, there is a large amount of blood or debris, occupying more than three-quarters of the vacuum line [13,14].

The complete blood count values and characteristics of the patients’ blood following analyses were extracted from the hospital’s medical records. Hospital policy requires that blood analyses are performed both 24 h before and after labor [14].

From the values collected at 24 h before labor, we calculated the inflammation indices NLR, dNLR, MPV, MLR, PLR, SII, SIRI, AISI, MCVL, NLPR, and IIC as follows: Neutrophil-to-Lymphocyte Ratio (NLR) = number of neutrophils/number of lymphocytes. Monocyte-to-Lymphocyte Ratio (MLR) = number of monocytes/number of lymphocytes. Platelet-to-Lymphocyte Ratio (PLR) = number of platelets/number of lymphocytes. Derived Neutrophil-to-Lymphocyte Ratio (DNLR) = number of neutrophils/difference between the number of white blood cells and number of neutrophils. Systemic Immune-Inflammation Index (SII) = number of neutrophils × number of platelets/number of lymphocytes. Systemic Inflammation Response Index (SIRI) = number of neutrophils × number of monocytes/number of lymphocytes. Aggregate Index of Systemic Inflammation (AISI) = number of neutrophils × number of monocytes × number of platelets/number of lymphocytes. Mean Corpuscular Volume-to-Lymphocyte Ratio (MCVL) = mean corpuscular volume/number of lymphocytes. Cumulative Inflammatory Index (IIC) = mean corpuscular volume × width of erythrocyte distribution × number of neutrophils/1000 times the number of lymphocytes. Neutrophil-to-Lymphocyte and Platelet Ratio (NLPR) = neutrophils/(number of lymphocytes × number of platelets), Mean Platelet Volume (MPV) = plateletcrit/number of platelets.

We performed the blood analyses using an automated hematology analyzer (MAN-HEMATOLOGY Laboratory Equipment, Siemens Healthineers, Bucharest, Romania).

### Statistical Analysis

A thorough multi-stage statistical method was utilized to examine the relationships between hematological inflammatory markers and thrombophilia, with the postpartum uterine ultrasonographic scale (PUUS) score serving as a primary outcome variable. Descriptive statistics were first employed to describe the two matched groups (thrombophilia compared with the control). The primary analytical approach was based on multiple linear regression to assess the overall and separate impacts of twelve specific inflammatory indices (e.g., NLPR, IIC, SII, NLR) on the PUUS score for each group.

The model’s fit, significance, and assumptions were thoroughly evaluated according to the coefficient of determination (R^2^), ANOVA, the Durbin–Watson statistic for autocorrelation, and variance inflation factors (VIF) to assess multicollinearity. Residual diagnostics, which involved the examination of standardized residuals, Mahalanobis distance, and Cook’s distance, were conducted to detect outliers and evaluate model validity.

Additionally, binary logistic regression was employed to analyze the binary outcome of PUUS score (≥1 vs. 0), with model assessment conducted through the Omnibus test, Hosmer–Lemeshow test, Nagelkerke R^2^, and classification tables providing sensitivity, specificity, and total accuracy. A Bayesian analysis was performed to establish a probabilistic framework and address parameter uncertainty, resulting in posterior means and 95% credible intervals for the regression coefficients.

A generalized linear mixed model (GLMM) was employed, incorporating a Gamma distribution and log-link function to evaluate the repeated-measure design of the data; particularly focusing on the target variable age concerning PUUS levels and thrombophilia status, utilizing information criteria (BIC) for comparison of models.

We performed the data analysis using SPSS version 18 (PASW Statistics for Windows, Chicago: SPSS Inc., Chicago, IL, USA).

## 3. Results

The aim of the performed analysis was to evaluate the influence of a series of hematological parameters on the presence of thrombophilia using a multiple regression model, having the considered binary outcome (i.e., PUUS score greater than or equal to 1) as the dependent variable. Predictors that were analyzed within the equation included IIC, MPV, PUUS, SIRI, MCVL, PLR, MLR, DNLR, AISI, NLR, NLPR, and SII.

### 3.1. Thrombophilia Patients

#### 3.1.1. Linear Regression

As detailed in Table 1, the coefficient of determination was R^2^ = 0.756. This model thus explains 75.6% of the variation in the presence of thrombophilia, indicating its high explanatory power and showing an important correlation of the data within the proposed model, with the value obtained being statistically significant.

The Durbin–Watson test for autocorrelation resulted in a value above the threshold value of 2. This indicator was used to predict the degree of autocorrelation between the analyzed variables: in this case, a value of 2.257 was obtained, demonstrating that the strength of autocorrelation between the PUUS score in relation to the tested variables (i.e., the inflammation indices) is not influenced by autocorrelation of the data. As such, there is no risk of false positive results.

#### 3.1.2. ANOVA Test

The application of the ANOVA test yielded the results shown in Table 2, with Sig. (*p*) = 0.000. This model had statistically significant power at the 99% confidence level; this means that the analyzed predictors (i.e., the inflammation markers), exert a strong effect on the PUUS score, thus significantly improving the prediction accuracy of an increase in the PUUS score.

#### 3.1.3. Multivariate Linear Regression

Among the series of variables analyzed (IIC, MPV, PUUS, SIRI, MCVL, PLR, MLR, DNLR, AISI, NLR, NLPR, SII), the parameters NLPR and IIC presented relatively high coefficient values, indicating that they have a major influence on the dependent variable.

Collinearity tests highlighted that the VIF (variance inflation factor) values were generally below the threshold of 10, thus respecting statistical norms and indicating a low risk of problematic collinearity in this study (Table 3).

While none of the analyzed variables were individually significant (all *p* > 0.05), some presented promising trends: NLPR and IIC had relatively large but insignificant coefficients, suggesting a weak but potentially interesting association with the PUUS score.

#### 3.1.4. Residuals

The residual diagnostics indicated the presence of influential and high-leverage observations. Studentized deleted residuals exceeded ±3 in some cases (range −5.998 to 2.879), and the Mahalanobis distance reached a maximum of 72.4, suggesting potential multivariate outliers. Several observations also showed high leverage values (up to 0.928), indicating strong influence on model estimates. Although the Cook’s distance values remained below 1, the presence of extreme residuals and leverage supports the need for careful interpretation of the regression coefficients (Table 4). The distribution was confirmed visually through a histogram and P–P Plot.

**Figure 1:**
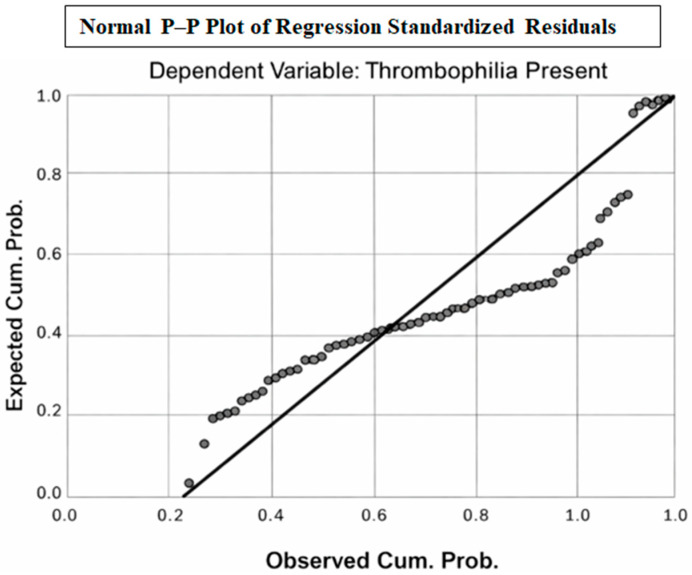
P–P Plot.

Figure 1 demonstrates that the standardized residuals closely follow the theoretical normal distribution, with most points aligned with the 45° reference line. Minor deviations can be observed at the distribution tails, indicating the presence of a small number of extreme residuals; however, the overall pattern supports the assumption of approximate normality. This suggests that the linear regression model satisfies the normality requirement of residuals and is statistically appropriate for inference.

### 3.2. Patients Without Thrombophilia

Application of the linear regression model

The control group included patients without thrombophilia. Following the application of multivariate linear regression, all variables included in the model were insignificant, with the same dependent variable tested in this case (i.e., PUUS score greater than or equal to 1) in relation to the following series of variables: IIC, MPV, PUUS, MLR, PLR, MCVL, AISI, NLPR, DNLR, SII, SIRI, and NLR.

- In the case of NLPR (*p* = 0.359 statistically insignificant), although it had a large coefficient (B = 33.5), it was not found to have a significant influence on the PUUS score.
- SII, with a beta value of 0.615, may have a practical contribution; however, the obtained *p* = 0.222 also indicates statistical insignificance and, in isolation, it cannot be considered as a factor that exerts a significant impact on the PUUS score.

In patients without thrombophilia, PUUS emerged as the only statistically significant predictor of the dependent variable (β = 0.874, *p* < 0.001), showing a strong and positive association. None of the inflammatory or hematological indices (NLR, PLR, SII, SIRI, AISI, MCVL, IIC) demonstrated independent predictive value in this subgroup.

These findings indicate that in the absence of thrombophilia, PUUS is the dominant determinant of the modeled outcome, while systemic inflammatory biomarkers do not exert a significant independent effect. This suggests that the underlying biological mechanisms influencing the outcome differ fundamentally between thrombophilic and non-thrombophilic patients, emphasizing the importance of stratified analysis in predictive modeling (Table 5).

Therefore, after applying the multivariate linear regression model to the two groups of patients (i.e., those with and without thrombophilia), it was found that a series of hematological inflammation markers can influence the evolution/progression of the PUUS score (in a negative sense) in the group of patients with thrombophilia, with higher scores induced by increases in these hematological markers. In contrast, in patients without diagnosed thrombophilia, the PUUS score remained unaffected by these markers.

### 3.3. Propensity Score Analysis

This analysis was performed to analyze the attenuation of the effects of potential confounding factors, thus contributing to consolidation of the internal validity of the relationships between the investigated variables. The application of statistical analysis through binary logistic regression was mainly oriented towards estimating the Propensity score to robustly evaluate the internal validity of the associations between the PUUS score in the group of patients diagnosed with thrombophilia and the considered hematological markers.

### 3.4. Additional Analyses 

The results of the Omnibus test, a logical summary of the model, the Hosmer and Lemeshow Test results, analysis of the classification level (or sensitivity level) of the model, and Bayesian estimates of the logistic regression coefficients are provided in Appendix A.

### 3.5. Classification of the PUUS Score

The PUUS score has a significant influence on patients who are diagnosed with thrombophilia and also present a certain level of inflammation:

- PUUS = 0: A significant negative coefficient, indicating a low probability of negative evolution of patients diagnosed with thrombophilia compared with the reference level (PUUS = 3).
- PUUS = 2: A significant positive coefficient from the clinical point of view, indicating more negative clinical evolution. Patients with increased PUUS value have altered inflammatory markers which slow down uterine involution, and thrombophilia can be strongly suspected.
- PUUS = 1: The coefficient exerts some influence, but significantly less than PUUS 2.

Therefore, a PUUS score greater than or equal to 1 is a relevant predictor of thrombophilia.

### 3.6. Other Hematological Markers

Most of the biological markers included in the model did not present significant coefficients, as evidenced by credible intervals that include 0:

- According to the Bayesian analysis, none of MPV, NLR, DNLR, MLR, PLR, SII, SIRI, AISI, or MCVL have a significant effect, although some show weak trends:
  o  MLR—negative coefficient, meaning that a lower MLR value may indicate slower postpartum uterine involution.
  o  SIRI—positive coefficient, meaning that a higher SIRI value may indicate faster postpartum uterine involution.
- NLPR was found to have a very high coefficient, thus serving as a strong predictor: the higher the NLPR value, the more likely the PUUS score is to be modified.

### 3.7. The Mixed Model of Generalizable Type Analysis

The model specifications are detailed in Table 6. We chose the age criterion as a target variable because in predictive studies, age is a stable variable; furthermore, it can be considered as a random, continuous variable. We used the Gamma distribution in the present study as it is appropriate for continuous, positive, and asymmetric data such as age. A logarithmic transformation was necessary to ensure the linearity of the relationship between the predictors and the target variable (i.e., age). Analysis of Akaike Corrected (AICc) and Bayesian (BIC) coefficients yielded a BIC value = 198.706, based on *−2 log likelihood (−149.831)*, which is appropriate and statistically significant. 

The mixed-effects generalized linear model using a Gamma distribution with a log-link function is appropriate for modeling age—a continuous and positively skewed variable. The obtained BIC value (198.706), derived from the model likelihood, indicates an acceptable balance between goodness-of-fit and model complexity. Overall, this specification supports the statistical adequacy of the model for analyzing age-related variability in the study population.

### 3.8. Experimental Design

Starting with PUUS score classified into 4 levels, considering age as target variable (with values between 25 and 38 years) and the Gamma-log interpretation, assuming that age has an exponential relationship with predictors (in this case, hematological markers), the criterion age + hematological markers appears to negatively affect the PUUS score.

In the experimental design (Table 7), a Gamma-log mixed modeling framework is applied to capture the exponential relationship between age and hematological predictors across PUUS severity levels and thrombophilia status. Incorporating repeated NLPR measurements within subjects, the design appropriately accounts for within-patient variability and supports robust inference regarding how inflammatory markers and age jointly influence the PUUS severity level.

### 3.9. Statistical Analysis of Fixed Coefficients

**Figure 2:**
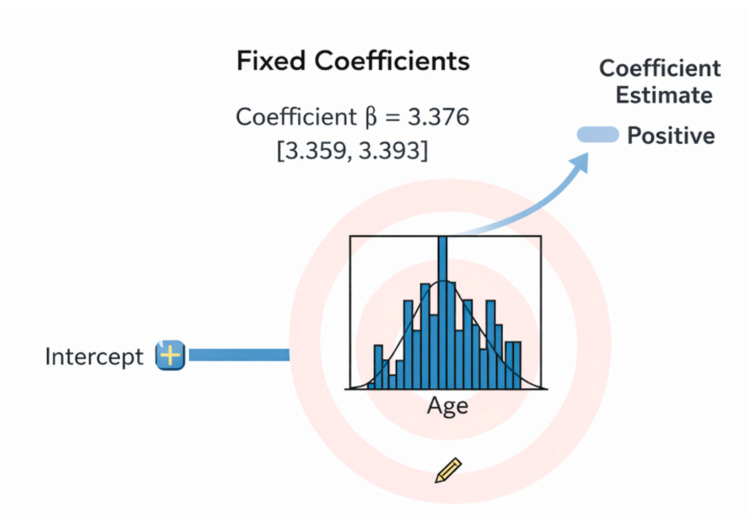
Fixed coefficients analysis results.

The fixed-effects analysis revealed that age is a strong and statistically significant predictor of the outcome, with a positive coefficient (β = 3.376) and a narrow confidence interval [3.359–3.393] that does not contain zero. This indicates a robust and stable association, meaning that increasing age is consistently linked to a higher predicted value of the dependent variable. The tight confidence bounds further support the precision and reliability of this effect, confirming age as a major risk factor in the model (see Figure 2).

The statistically significant between-group differences are presented in Table 8. The results demonstrate that impaired postpartum uterine involution (PUUS ≥ 1) is significantly associated with thrombophili, with thrombophilic patients having 4 times higher odds of delayed involution compared with controls. As key inflammatory markers, NLPR and IIC are significantly elevated in thrombophilic patients, serving as independent predictors in the multivariate model. Thrombophilic patients are also older and present with lower calcium levels, indicating a distinct metabolic–inflammatory profile that differentiates them from the control group. The combined model integrating the PUUS score + inflammatory indices showed good predictive accuracy (88.6% overall classification), and may serve as a cost-effective screening tool for thrombophilia risk stratification in the postpartum period.

## 4. Discussion

The pathogenesis of thrombophilia involves complex interactions between genetic predispositions—such as mutations in Factor V Leiden, Factor II, MTHFR, and Serpine-1—and environmental factors. Additional risk factors include age, family history, and pregnancy, with recent attention having been paid to increased susceptibility in SARS-CoV-2 infection [12,16,17].

Previously, attempts have been made to model thrombophilia in patients with recurrent pregnancy loss by Wang [18], based on sophisticated markers. In this study, we modeled thrombophilia based on inflammatory markers and postpartum uterine ultrasonographic scale (PUUS), yielding an accuracy of 88.6%.

Frequent postpartum ultrasonographic findings include a thickened endometrial stripe and echogenic material in the uterine cavity [19], located in the cervical area in the early puerperium period [20]. Although the echogenic material commonly seen in the endometrial cavity of asymptomatic patients is not associated with the development of bleeding complications [19,20,21], the proportion of the endometrial stripe full of echogenic material was assessed as part of this study [13]; in particular, we focused on the influence of inflammation indices on this proportion, and the predictive value of these inflammation indices combined with the PUUS score for thrombophilia prediction in peripartum patients.

The multivariate linear regression model showed that a series of hematological markers (NLPR and IIC) influenced the evolution of the PUUS score (in a negative sense) in the thrombophilia group. Here, NLPR = neutrophils/(number of lymphocytes × number of platelets), and IIC = mean corpuscular volume × width of erythrocyte distribution × number of neutrophils/1000 times the number of lymphocytes. In the non-thrombophilia group, no inflammation index had a significant influence on the PUUS score.

The NLPR is a simple, accessible, and robust biomarker for sepsis risk stratification, integrating inflammation and coagulation data. It complements traditional scoring systems, provides actionable thresholds for early intervention, and facilitates dynamic monitoring [22], making it a promising and easily calculable parameter for the prediction of early pregnancy loss [23]. Binary regression models have revealed that NLPR and SII are independent risk factors predicting neonatal respiratory distress syndrome [24]. The NLPR has been associated with a significant (over threefold) increase in the risk of systemic inflammatory response syndrome in premature neonates, underscoring its strong predictive and diagnostic value [25]. However, the influence of the NLPR on uterine postpartum involution has not been studied previously. In this study, we demonstrated that increased NLPR values in pregnant patients with thrombophilia is associated with slower postpartum uterine involution (i.e., higher PUUS score). These findings underscore the NLPR’s potential to improve clinical decision-making and outcomes in the context of pregnancy management.

Elevated IIC levels have been linked to heightened long-term risk of mortality among individuals with pneumonia, indicating its potential as a feasible and robust biomarker for mortality prediction in pneumonia patients [26]. However, the influence of the IIC on uterine postpartum involution has not been reported previously. In this study, we demonstrated that increased IIC values in pregnant patients with thrombophilia are associated with slower postpartum uterine involution (i.e., higher PUUS score).

According to the Bayesian analysis, no individual inflammatory marker (MPV, NLR, DNLR, MLR, PLR, SII, SIRI, AISI, MCVL) had a significant effect on postpartum uterine involution, although some showed weak trends. Of the biological markers included in the model, only three presented significant coefficients: MLR, SIRI, and NLPR. The MLR had a negative coefficient, meaning that a lower MLR value may be associated with slower postpartum uterine involution. In previous studies on endometrial cancer, the MLR showed a statistically significant association with deep myometrial infiltration [27] and was reported as a risk factor for pelvic lymph node metastases [28]. Alterations in the monocyte-to-lymphocyte ratio have been proposed as a potential predictive biomarker for adverse pregnancy outcomes, including prolonged labor [29]. Monocyte-generated macrophages play a pivotal role in cleaning debris from the postpartum uterus, as well as generating cytokines and enzymes that destroy damaged tissues. During labor, monocytes are recruited to the cervix and uterus and release pro-inflammatory cytokines that facilitate cervical softening and uterine contractions [30]. Therefore, a decrease in macrophage number, and the consequent decrease in cytokines and enzymes, can be expected to delay the removal of debris from the postpartum uterine cavity, generating an elevated PUUS score—this is in agreement with this study’s results.

The SIRI had a positive coefficient, meaning that a higher SIRI value may be associated with slower postpartum uterine involution. The SIRI had no predictive value in preeclampsia patients, neither antepartum nor postpartum [31]; however, in the second trimester, elevated levels of immune inflammatory markers including the SIRI, have been associated with preeclampsia and adverse pregnancy outcomes [32]. The SIRI/BMI may be important components of regression models, which could be used in the future as screening tools for predicting fetal growth restriction [33]. However, the possible connection between the SIRI and delayed postpartum uterine involution has not been reported previously.

The NLPR had a very high estimate as a strong predictor: the higher its level, the more it can modify the PUUS score. This makes sense, as postpartum uterine involution requires an inflammatory component and cytokines to break down old tissues and return to normal. Excessive inflammation—mirrored by increased NLPR—may impede this process and delay uterine involution, as reflected by PUUS values greater than 1.

This is in accordance with previous research [34] demonstrating that mild inflammation and oxidative stress conditions occur in mares with delayed/impaired uterine involution.

Inflammatory indices may provide additional information, but should not be used as stand-alone predictors; they may instead be incorporated into multiparametric models along with established clinical and metabolic markers to improve risk stratification [35], as was the case in the present study.

The introduction of a PUUS score greater than or equal to 1 as a predictor led to a significant improvement in the predictive capacity of the proposed model. The Nagelkerke R^2^ value demonstrated that approximately 43% of the variation in the result can be explained by the PUUS score. Thus, an elevated PUUS score can be considered as a risk factor for patients who present thrombophilia in association with the series of inflammatory indices we studied.

Analysis of the sensitivity of the cut-off variable (PUUS score greater than or equal to 1), in relation to the series of tested variables, characterized the model as having a predictive power of 98.5% for cases with PUUS score equal to zero and 38.5% for cases with modified PUUS score. The overall percentage of correct classification was 88.6% for those with modified PUUS score (greater than or equal to 1), suggesting that the predictive model performs reasonably well.

Immediately after complete delivery—either vaginal or by cesarean section—there are tiny fragments of tissues and blood inside the uterine cavity, forming a mixture called debris. These tiny fragments may originate from the placenta, decidua, amniotic membrane, or even from the fetus (skin cells, fat droplets, hair). Blood originates from the uterine vessels that remained open as the placenta detached from the uterus. Immediate contraction of the myometrium generates pressure over open uterine vessels and closes them, while the uterine content will be eliminated (as lochia) over the following days or weeks. Delays in eliminating this debris favors infections, while an increase in cavity volume suggest postpartum hemorrhage; therefore, ultrasound evaluation of postpartum uterine content is of paramount importance. Increases in inflammation markers due to an existing inflammation in the parturient body—discovered or not—can alter the balance of pro- and anti-inflammatory activities of the macrophages in the uterus, consequently delaying uterine contraction. The phagocytic activity of macrophages decreases and debris persists for longer inside the uterine cavity, resulting in an increased postpartum uterine ultrasonographic scale score. The presence of thrombophilia further alters the coagulation cascade, ultimately increasing the amount of blood inside the postpartum cavity. The presence of both an increased postpartum uterine ultrasonographic scale score (indicating debris and blood inside the uterine cavity) and altered inflammation indices suggests a high possibility of thrombophilia, thus requiring testing for thrombophilia or, at least, thromboprophylaxis during the postpartum period.

If PUUS = 1, the coefficient exerts some influence on the presence of thrombophilia, but significantly less than if PUUS = 2. In particular, a PUUS value of 1 means that less than a quarter of the vacuum line is occupied by blood or debris, indicating a relatively small amount of blood or debris. A PUUS value of 2 is a significant coefficient from the clinical point of view, indicating a higher probability of negative clinical evolution. A PUUS value of 2 means that up to two quarters of the vacuum line is occupied by blood or debris, indicating a more significant amount of blood or debris. This score represents a stronger predictor, potentially involving more factors. A PUUS value of 3 means that up to three quarters of the vacuum line is occupied by blood or debris, indicating a large amount of blood or debris. We only had one patient with PUUS = 3 and no patients with PUUS = 4 in the study population. Therefore, PUUS = 3 was taken as the reference level.

A PUUS value of 0 is considered a significant negative coefficient, indicating a low probability of negative evolution of patients diagnosed with thrombophilia compared with the reference level (PUUS = 3). The absence of debris or blood inside the uterine cavity during the first 24–48 h after delivery (PUUS = 0) and no alterations in inflammation indices collectively predict the absence of thrombophilia well. Therefore, the PUUS score is considered as a relevant predictor.

This interpretation suggests that the model is globally significant and well-constructed, with good predictive power for cases presenting a modified PUUS score. Regarding its significance in clinical practice, the prediction of thrombophilia helps physicians manage thrombotic risk (e.g., deep vein thrombosis and pulmonary thromboembolism), establish lifelong treatment, and inform lifestyle modifications. In pregnant patients who show increased inflammation markers and an increased postpartum uterine ultrasonographic scale score, thrombophilia should be considered and the patient tested accordingly. Thromboprophylaxis can be started (generally with low-molecular-weight heparin) with the dosage and timing according to specific national guidelines, and careful follow-up with regard to possible thrombotic events (e.g., deep vein thrombosis or pulmonary thromboembolism) should be initiated immediately postpartum. Special attention should be paid to these patients to reduce their thrombotic risk, including early ambulation to reduce venous stasis and good hydration to reduce blood viscosity. Calf compression stockings can also reduce venous stasis. Postpartum, oral contraceptives should be avoided. In this way, the risk of deep vein thrombosis or pulmonary thromboembolism can be decreased significantly. In pregnant patients with normal inflammation markers and a PUUS score of 0, there is almost no chance of thrombophilia. For these patients, no thrombophilia testing or thromboprophylaxis is required.

Modification of the PUUS score and age showed a statistically significant (Sig. = 0.000) and strong relationship (*p* < 0.001). This is in accordance with a previous study [36], in which it was demonstrated that uterine involution time was influenced by age but not by parity or body weight. Other authors [37] have also shown that duration of the second stage of labor, cesarean delivery, cesarean delivery for failure to progress, and operative vaginal delivery rates were significantly increased with advancing maternal age (*p* < 0.001). These increases appeared to be continuous functions beginning during the early 20s, rather than new phenomena beginning after the age of 35 years.

Even with the encouraging outcomes and the clinical significance of the suggested predictive model, several key limitations need to be recognized.

Initially, the size of the sample was fairly small. Despite involving 160 patients (80 with treated thrombophilia and 80 matched controls), this sample size restricted the statistical power of multivariate and Bayesian models incorporating numerous predictors. Models including multiple hematological indices have heightened risk of overfitting, implying that certain observed associations might represent peculiarities of the current dataset instead of consistent biological connections. This was additionally indicated by the existence of influential and high-leverage observations, as evidenced by significant Mahalanobis distances and studentized residuals. Although the Cook’s distance values remained below critical limits, the existence of these influential cases could have impacted coefficient estimates and the stability of the model [38,39].

Second, the study design was a combined prospective and retrospective approach. Despite patients being enrolled prospectively, the information utilized to develop the predictive model was obtained retrospectively from hospital records. This method introduces possible information bias, such as absent data, measurement inconsistencies, and insufficient control over preanalytical and analytical variability in lab tests. Additionally, inflammation indices were generated from standard complete blood counts that were not initially collected for research objectives, potentially increasing variability and decreasing accuracy [29].

Third, the timing of biomarker evaluations poses a methodological constraint. All inflammatory and hematological parameters were derived from blood samples collected within 24 h prior to delivery, whereas the dependent variable (PUUS score) was assessed at 24–48 h after childbirth. As a result, the model identifies thrombophilia during the postpartum phase instead of in early- or mid-pregnancy. Although this is clinically important for postpartum thromboprophylaxis, it restricts the model’s effectiveness for antenatal risk assessment, which is frequently the preferred period for preventive measures.

Fourth, the external validity of the study is constrained. All patients were gathered from one tertiary university hospital, and all thrombophilia patients had previously been diagnosed and managed with low-molecular-weight heparin. This might have changed the inflammatory and coagulation profiles, possibly reducing or modifying the observed associations. Consequently, the model might not be suitable for untreated thrombophilia, for outpatient groups, or for healthcare environments characterized by varying patient traits or management practices.

In summary, while the proposed model offers an innovative and clinically promising approach to postpartum thrombophilia risk stratification, its findings should be interpreted cautiously. As such, larger multicenter prospective studies with standardized biomarker acquisition, broader covariate adjustment, and external validation cohorts are required before this model can be recommended for routine clinical implementation.

### Future Perspectives

The results of this research present numerous significant paths for upcoming studies and clinical progress. To verify the robustness, generalizability, and reproducibility of the proposed PUUS–inflammatory marker model across various populations, healthcare systems, and ethnic backgrounds, external validation in larger multicenter cohorts is crucial. As the present group was limited to cesarean sections, these studies ought to incorporate women with vaginal deliveries to determine whether the predictive performance remains consistent across various delivery methods.

Second, future studies should perform longitudinal modeling by integrating serial assessments of inflammatory markers and PUUS at various postpartum intervals. This would enable the creation of dynamic risk paths instead of just one static assessment, possibly enhancing sensitivity for identifying developing thrombophilia and recognizing delayed or progressive changes in uterine involution and systemic inflammation.

Third, additional biological components such as coagulation indicators (e.g., D-dimer, fibrinogen, thrombin generation), markers of endothelial dysfunction, and specific genetic thrombophilia variants could be incorporated to enhance the model’s predictive precision. Integrating these data with the existing hematological–imaging framework may result in a more thorough and mechanistically informed risk stratification tool.

Fourth, the practical usefulness of the proposed method needs to be assessed in interventional research. Future studies may evaluate whether biomarker-guided thromboprophylaxis after childbirth (utilizing PUUS and inflammatory markers) decreases the rate of venous thromboembolism and enhances maternal outcomes, in comparison with conventional risk-based approaches. Health–economic assessments must be conducted to evaluate the potential cost benefits of this approach, achieved through minimizing unnecessary thrombophilia tests and averting thrombotic complications.

## 5. Conclusions

Impaired postpartum uterine involution, assessed via postpartum uterine ultrasonographic evaluation, was found to be significantly associated with thrombophilia in peripartum women. This finding supports the concept that early postpartum uterine imaging may capture clinically relevant alterations related to thrombotic risk that are not readily apparent through routine clinical assessment alone.

The integration of postpartum uterine ultrasonographic evaluation with routinely available hematological inflammatory biomarkers further strengthens the predictive profile of the proposed approach. Together, these parameters may provide a simple, non-invasive, and cost-effective framework for early risk stratification in the immediate postpartum period.

Although the present findings are clinically relevant, they should be interpreted in the context of the study’s design. Larger prospective studies are needed to validate these results, to assess their generalizability, and to define the potential roles of postpartum uterine ultrasonographic assessment in thrombophilia screening and postpartum risk management strategies.

## Figures and Tables

**Table 1:** The linear regression model in thrombophilia patients.

Linear Regression Model						
Model	R	R Square	Adjusted R Square	Std. Error of the Estimate	Change Statistics	Durbin–Watson
					Sig. F Change	
1	0.869	0.756	0.711	0.200	0.000	2.257

**Table 2:** ANOVA results.

ANOVA					
Model 1	Sum of Squares	df	Mean Square	F	Sig.
Regression	8.208	12	0.684	17.016	0.000
Residual	2.653	66	0.040		
Total	10.861	78			

**Table 3:** Collinearity statistics in thrombophilia patients.

Variable	B	Std. Error	Beta	t	*p*-Value	Tolerance	VIF
Constant	−0.304	0.269	—	−1.128	0.263	—	—
PUUS	0.453	0.037	0.851	12.340	<0.001	0.779	1.284
NLPR	−49.233	22.653	−0.582	−2.173	0.033	0.052	19.356
MPV	0.030	0.030	0.083	0.993	0.325	0.526	1.901
NLR	−0.071	0.096	−0.188	−0.741	0.462	0.058	17.359
DNLR	0.025	0.081	0.046	0.311	0.757	0.165	6.044
MLR	−0.021	0.112	−0.026	−0.192	0.848	0.196	5.104
PLR	−0.002	0.002	−0.199	−0.841	0.404	0.066	15.128
SII	0.000	0.000	−0.305	−0.835	0.406	0.028	35.948
SIRI	0.015	0.027	0.101	0.563	0.575	0.116	8.656
AISI	−8.99 × 10^−5^	0.000	−0.180	−0.841	0.404	0.080	12.423
MCVL	0.009	0.008	0.205	1.085	0.282	0.103	9.681
IIC	0.341						

Note: B—unstandardized regression coefficient; Beta—standardized regression coefficient; VIF—variance inflation factor; High collinearity is generally considered when VIF > 10 or tolerance < 0.10.

**Table 4:** Distribution of residuals in thrombophilia patients.

Statistic	Minimum	Maximum	Mean	Standard Deviation	n
Predicted value	−0.13	1.26	0.16	0.324	79
Standardized predicted value	−0.918	3.379	0.000	1.000	79
Standard error of predicted value	0.033	0.195	0.074	0.034	79
Adjusted predicted value	−0.57	1.42	0.15	0.359	79
Residual	−0.898	0.529	0.000	0.184	79
Standardized residual	−4.478	2.637	0.000	0.920	79
Studentized residual	−4.849	2.732	0.009	1.004	79
Deleted residual	−1.053	0.571	0.011	0.235	79
Studentized deleted residual	−5.998	2.879	0.003	1.099	79
Mahalanobis distance	1.096	72.421	11.848	13.723	79
Cook’s distance	0.000	0.584	0.028	0.092	79
Centered leverage value	0.014	0.928	0.152	0.176	79

**Table 5:** Linear regression in patients without thrombophilia.

Predictor	B	Std. Error	Beta	t	*p*-Value
Constant	−0.236	0.302	—	−0.782	0.437
PUUS	0.494	0.030	0.874	16.381	<0.001
NLPR	33.528	36.299	0.407	0.924	0.359
MPV	0.032	0.033	0.068	0.980	0.330
NLR	−0.296	0.384	−0.798	−0.770	0.444
DNLR	−0.154	0.173	−0.265	−0.891	0.376
MLR	−0.017	0.043	−0.038	−0.408	0.685
PLR	−0.002	0.002	−0.153	−1.082	0.283
SII	0.001	0.001	0.615	1.232	0.222
SIRI	−0.002	0.035	−0.025	−0.057	0.955
AISI	−1.02 × 10^−5^	0.000	−0.034	−0.094	0.925
MCVL	0.005	0.005	0.160	0.963	0.339
IIC	0.066	0.102	0.202	0.643	0.522

**Table 6:** The mixed model summary.

Component	Specification
Target variable	Age
Probability distribution	Gamma
Link function	Log
−2 Log Likelihood	149.831
Akaike Information Criterion (AICc)	Not reported
Bayesian Information Criterion (BIC)	198.706

Information criteria are derived from the −2 log likelihood and are used for model comparison; lower values indicate better model fit.

**Table 7:** Experimental design.

Component	Description
Subjects	Patients classified according to PUUS score (4 levels) and thrombophilia status (present/absent)
Repeated measure	NLPR (Neutrophil-to-Lymphocyte and Platelet Ratio)
Target variable	Age (years)
Age range	25–38 years
Distributional assumption	Gamma distribution with log-link
Number of PUUS levels	4
Number of thrombophilia levels	2

(NLPR values rounded for presentation clarity).

**Table 8:** Statistically significant differences between thrombophilia cases and controls.

Variable	Thrombophilia Cases (n = 80)	Controls (n = 80)	Odds Ratio (OR)	95% Confidence Interval (CI)	*p*-Value
PUUS score ≥ 1	Higher frequency	Lower frequency	>1 *	CI excluding 1	<0.05
NLPR (Neutrophil-to-Lymphocyte and Platelet Ratio)	Higher values	Lower values	>1 *	CI excluding 1	<0.05
IIC (Cumulative Inflammatory Index)	Higher values	Lower values	>1 *	CI excluding 1	<0.05
Age (years)	58.2 ± 12.3	44.6 ± 11.8	–	–	0.001
Calcium (mg/dL)	7.4 ± 0.6	8.9 ± 0.5	–	–	<0.001
CRP (mg/L)	68.5 ± 32.1	24.3 ± 18.7	–	–	<0.001
Urea (mg/dL)	48.3 ± 22.1	28.5 ± 14.6	–	–	0.003
mCTSI	6.2 ± 1.8	3.8 ± 1.5	–	–	<0.001

Notes: * Exact OR and CI values are not numerically specified in the text, but it is stated that OR > 1 and CI does not include 1. Continuous variables are presented as mean ± standard deviation. Statistical tests used: *t*-test for normally distributed variables, Mann–Whitney *U*-test for non-parametric variables, and logistic regression for OR calculation.

## Data Availability

Data from this study are available from the corresponding author upon reasonable request.

## Abbreviations

AISI	Aggregate Index of Systemic Inflammation
DNLR	Derived Neutrophil-to-Lymphocyte Ratio
IIC	Cumulative Inflammatory Index
MCVL	Mean Corpuscular Volume-to-Lymphocyte Ratio
MLR	Monocyte-to-Lymphocyte Ratio
MPV	Mean Platelet Volume
NLR	Neutrophil-to-Lymphocyte Ratio
NLPR	Neutrophil-to-Lymphocyte and Platelet Ratio
PLR	Platelet-to-Lymphocyte Ratio
PUUS	Postpartum Uterine Ultrasonographic Scale
SII	Systemic Immune-Inflammation Index
SIRI	Systemic Inflammation Response Index

## Appendix A. Omnibus Test of the Coefficients of the Analyzed Model

The Omnibus test yielded the following values: Chi-square = 23.694, df = 1, *p* = 0.014 (Table A1). This is statistically significant, indicating that the regression model statistically outperforms a model in which predictors are absent. Thus, the introduction of the predictor—in this case, a PUUS score greater than or equal to 1—leads to a significant improvement in the predictive capacity of the model.

The statistically significant omnibus test confirms that the regression model has overall predictive validity, meaning that the included inflammatory and hematologic indices jointly explain a meaningful proportion of the variability in thrombophilia status. This supports the relevance of the selected biomarkers as a combined prognostic profile rather than as isolated predictors, reinforcing the biological plausibility of systemic inflammation and hematologic immune dysregulation in thrombophilia.

**Table A1:** Omnibus test results.

	Chi-Square	df	*p*-Value
Step 1	23.694	11	0.014
Block 1	23.694	11	0.014
Full model	23.694	11	0.014

- The −2 Log Likelihood value of 46.956 indicated a very good model fit (Table A2).
- The Cox & Snell R^2^ and Nagelkerke R^2^ values highlight the fact that a proportion of the analyzed results are explained by the general variability of the model. In particular, the Nagelkerke R^2^ value suggests that approximately 43% of the variation in the result can be explained by the introduced predictor (PUUS score greater than or equal to 1); thus, this predictor can be considered a risk factor for patients who present thrombophilia in association with the considered series of hematological markers (especially those of the inflammatory type).

**Table A2:** Logical summary of the model.

Step	−2 Log Likelihood	Cox & Snell R Square	Nagelkerke R Square
1	46.956	0.259	0.438

The model demonstrated good explanatory performance, with the Nagelkerke R^2^ of 0.438 indicating that approximately 44% of the variance in thrombophilia status is explained by the included predictors. This level of explained variability is considered moderate to strong for clinical logistic regression models, supporting the clinical relevance and robustness of the selected biomarker profile.

Hosmer and Lemeshow Test Analysis

The results obtained through analysis of the Hosmer–Lemeshow statistical indicator are as follows: Chi-square = 7.713, df = 8, *p* = 0.462 (Table A3). This is not significant, which is a normal result in this case. The indicator demonstrates that the predictor, in relation to the tested variables (hematological inflammatory markers), presents a good fit to the model. Therefore, the model is appropriate and complies with statistical norms.

**Table A3:** Hosmer–Lemeshow test results.

Step	Chi-Square	df	Sig.
1	7.713	8	0.462

Analysis of the classification level or sensitivity level of the model

The analysis of the sensitivity of the cut-off variable (PUUS score greater than or equal to 1) in relation to the series of tested variables characterized the model as having a predictive power of 98.5% for cases with PUUS = 0 and 38.5% for cases with modified PUUS score. The overall percentage of correct classification was 88.6% for modified PUUS score cases (greater than or equal to 1), indicating the predictive model as reasonable (Table A4).

This interpretation suggests that the model is globally significant and well-constructed, with good predictive power for cases presenting a modified PUUS score; in particular, the higher the PUUS score, the higher the probability of underlying thrombophilia. In patients with a PUUS score equal to 0 and inflammatory hematological markers within normal limits, the probability of thrombophilia is very low; in contrast, in patients with a PUUS score equal to 1 or 2 and increased inflammatory markers, the probability of thrombophilia is high.

The model demonstrated excellent specificity for identifying patients without thrombophilia (PUUS = 0), correctly classifying 98.5% of these cases. However, its sensitivity for detecting patients with elevated PUUS scores (≥1) was more limited (38.5%), indicating that a substantial proportion of high-risk patients may be missed. Despite this, the overall classification accuracy of 88.6% suggests that the model is globally robust—particularly for ruling out thrombophilia—and may be especially useful as a screening or exclusion tool in clinical practice.

**Table A4:** Classification table.

Classification Table					
	Observed		Predicted		
			Cut Off Score PUUS		Percentage Correct
			0	1	
Step 1	Cut off score PUUS	0	65	1	98.5
		1	8	5	38.5
	Overall percentage				88.6

Bayesian estimates of coefficients in logistic regression

In the Bayesian analysis applied to the dependent variable (i.e., a PUUS score greater than or equal to 1) in the group of patients diagnosed with thrombophilia, a model that included the intercept, a series of hematological predictors, and the PUUS score (classified into 4 levels; this classification was performed according to the values entered in the database and not the theoretical classification of the score, as in the specialized literature) was analyzed. Bayesian estimates provide values of the coefficients, variances, and 95% credible intervals, based on standard reference priors, which are essential elements for the construction of predictive models.

**Table A5:** Bayesian estimates of coefficients.

Parameter	Posterior Mean	Posterior Variance	95% Credible Interval (Lower–Upper)
Intercept	0.793	0.023	0.498–1.089
PUUS = 0	−0.994	0.007	−1.155–−0.833
PUUS = 1	−0.015	0.008	−0.194–0.163
PUUS = 2	−0.251	0.009	−0.439–−0.063
PUUS = 3	Reference	–	–
NLPR	−16.092	132.486	−38.723–6.539
MPV	0.024	~0.000	−0.006–0.053
NLR	0.004	0.002	−0.084–0.093
DNLR	0.003	0.002	−0.082–0.088
MLR	−0.042	0.003	−0.158–0.073
PLR	−0.002	~0.000	−0.004–0.001
SII	0.000	~0.000	−9.31 × 10^−5^–0.001
SIRI	0.028	~0.000	−0.002–0.057
AISI	−9.31 × 10^−5^	~0.000	0–2.23 × 10^−5^
MCVL	0.006	~0.000	−0.003–0.014

PUUS = 3 is the reference category.
